# Brain Circuitries Involved in Sleep Disorders

**DOI:** 10.3389/fneur.2015.00066

**Published:** 2015-04-14

**Authors:** Anne-Marie Landtblom, Maria Engström

**Affiliations:** ^1^Department of Clinical and Experimental Medicine/Neurology, University of Linköping, Linköping, Sweden; ^2^Department of Neurology, Uppsala University, Uppsala, Sweden

**Keywords:** imaging, neuronal, narcolepsy, Kleine–Levin syndrome, hypersomnia, obstructive sleep apnea, pharmacotherapy, side effects

Sleep disorders are of increasing importance in the society and this is due to many reasons. Firstly, sleep disorder/disturbance in the young can have psychosocial consequences with an impact on education, work, and mental health, and may also underpin unemployment. One may suspect that the use of social media and nightly entertainment in young subjects may blur the clinical picture, and sometimes hinder a diagnosis of a real sleep disorder. Secondly, the impact of sleep disturbances is increasingly focused in the society with regard to traffic safety questions, where accidents can be related to reduced mental concentration due to sleepiness (1). Finally, the impact of sleep disturbance on the general outcome of a variety of diseases is increasingly highlighted, like for example in epilepsy, Alzheimer’s, and Parkinson’s disease (2).

Sleep disorders can be hard to diagnose: they often have an onset in young ages, but young people are on the other hand also prone to a physiological sleepiness or delayed sleep phase, sometimes facilitated by the use of social media and nightly entertainment. The latter circumstances can hinder the diagnosing, which sometimes is difficult and needs laboratory support (3). In addition, the young cases with narcolepsy due to the Pandemrix vaccination against swine flu in Europe in 2009/2010 indeed put a focus on sleep disorders as a whole, with increasing awareness among physicians but also in the society, and an increasing research activity (4–6). This situation indicated clearly that narcolepsy is an autoimmune disease and in fact recent research has clarified the pathology to a big extent: the experience with the Pandemrix/H1N1-initiated cases gave evidence that there is a combined pathogenesis with a genetic component (HLA DQB1*0602) and an environmental factor like vaccination/virus working through molecular mimicry. The harmed site is a neuronal locus in the lateral hypothalamus, where hypocretin-producing cells are severely affected. The etiology and mechanisms of other sleep disorders like, for example, hypersomnias are far more unclear. The rare form of periodic hypersomnia, the Kleine–Levin syndrome (KLS), is important to recognize, since it can be mixed up with physiological sleepiness in teenagers or psychiatric conditions, again pointing at the importance of a proper differential diagnosing (3, 7–9). Another condition of particular interest for the young is the delayed sleep phase syndrome (DSPS) (3). Finally, in many countries, there is increasing interest to detect and treat the obstructive sleep apnea syndrome (OSAS), since it is a condition with substantial impact on the occupational life and also with severe cardiovascular risks in the long run (10).

*This research topic specifically aims at describing the progress of imaging sciences in sleep medicine. It is a rapidly developing instrument, both regarding pathological and physiological conditions in humans*.

Today’s knowledge of sleep physiology is founded on observations that are about 100 years old: the key components of the sleep–wake regulatory systems originates from von Economo’s studies on a specific hypersomnia disorder, encephalitis lethargica. Already in the early twentieth century, von Economy identified the specific areas of the brain where lesions caused abnormal sleep–wake behavior. Quite recently, these discoveries have been actualized and more details of the brain circuitries and the neurotransmitters involved in sleep–wake regulation have been described. Nevertheless, the neural substrates that regulate our diurnal regulation of sleep and wakefulness are not yet completely understood. Here, Larson-Prior and co-workers review the present knowledge about brain circuits involved in sleep and sleep disorders (11). In addition, the authors address the important relation between sleep and cognition.

Sleep disorders can provide novel insights into the brain’s sleep–wake regulatory systems. Narcolepsy, which is characterized by sudden sleep attacks that can appear several times a day, is related to loss of hypocretin-producing neurons in the hypothalamus. The hypothalamus is one important node of one of the two major branches of the ascending arousal system. The other branch involves the thalamus, which might be affected in KLS. In a perspective article, Engström et al. suggest that functional magnetic resonance imaging (fMRI) can identify anatomical biomarkers of brain function related to narcolepsy and KLS and their concomitant symptoms (12). An interesting example is given in the case study that shows reduced thalamic and pontine connectivity during a hypersomnia period in KLS (13). A possible diagnostic method, single photon emission computed tomography (SPECT), is evaluated in the article by Vigren et al. (14).

Neural circuits involved in sleep disorders could also be studied by pharmacological interventions. Sveinsson discusses this topic in his case study on lithium treatment in KLS (15), and Sarkanen and co-workers report on psychosis in narcolepsy patients treated by sodium oxybate (16). Another intervention, continuous positive airway pressure (CPAP) in OSAS, is reviewed by Huynh and co-workers, interestingly using a radiological volumetric outcome (17).

A general clinical background is sketched in *The Sleepy Teenager* by Landtblom and Engström (3), aiming at building a structure for efficient diagnosing. Finally, a book review of *Neuroimaging of Sleep and Sleep Disorders* (18) concerning imaging methods in sleep medicine highlights the evolving investigation possibilities, from clinical and research aspects. This is a volume that we do recommend as a guide and inspiration.

## Conflict of Interest Statement

The authors declare that the research was conducted in the absence of any commercial or financial relationships that could be construed as a potential conflict of interest.
